# An improved genome assembly of the fluke *Schistosoma japonicum*

**DOI:** 10.1371/journal.pntd.0007612

**Published:** 2019-08-07

**Authors:** Fang Luo, Mingbo Yin, Xiaojin Mo, Chengsong Sun, Qunfeng Wu, Bingkuan Zhu, Manyu Xiang, Jipeng Wang, Yi Wang, Jian Li, Ting Zhang, Bin Xu, Huajun Zheng, Zheng Feng, Wei Hu

**Affiliations:** 1 Department of infectious diseases, Huashan Hospital, State Key Laboratory of Genetic Engineering, Ministry of Education Key Laboratory for Biodiversity Science and Ecological Engineering, Ministry of Education Key Laboratory of Contemporary Anthropology, School of Life Sciences, Fudan University, Shanghai, China; 2 National Institute of Parasitic Diseases, Chinese Center for Disease Control and Prevention, Key Laboratory of Parasite and Vector Biology of China Ministry of Health, WHO Collaborating Centre for Tropical Diseases, Joint Research Laboratory of Genetics and Ecology on Parasite-host Interaction, Chinese Center for Disease Control and Prevention & Fudan University, Shanghai, China; 3 Shanghai-MOST Key Laboratory of Health and Disease Genomics, Chinese National Human Genome Center at Shanghai, Shanghai, China; PUCRS, BRAZIL

## Abstract

**Background:**

*Schistosoma japonicum* is a parasitic flatworm that causes human schistosomiasis, which is a significant cause of morbidity in China and the Philippines. A single draft genome was available for *S*. *japonicum*, yet this assembly is very fragmented and only covers 90% of the genome, which make it difficult to be applied as a reference in functional genome analysis and genes discovery.

**Findings:**

In this study, we present a high-quality assembly of the fluke *S*. *japonicum* genome by combining 20 G (~53X) long single molecule real time sequencing reads with 80 G (~ 213X) Illumina paired-end reads. This improved genome assembly is approximately 370.5 Mb, with contig and scaffold N50 length of 871.9 kb and 1.09 Mb, representing 142.4-fold and 6.2-fold improvement over the released WGS-based assembly, respectively. Additionally, our assembly captured 85.2% complete and 4.6% partial eukaryotic Benchmarking Universal Single-Copy Orthologs. Repetitive elements account for 46.80% of the genome, and 10,089 of the protein-coding genes were predicted from the improved genome, of which 96.5% have been functionally annotated. Lastly, using the improved assembly, we identified 20 significantly expanded gene families in *S*. *japonicum*, and those genes were primarily enriched in functions of proteolysis and protein glycosylation.

**Conclusions:**

Using the combination of PacBio and Illumina Sequencing technologies, we provided an improved high-quality genome of *S*. *japonicum*. This improved genome assembly, as well as the annotation, will be useful for the comparative genomics of the flukes and more importantly facilitate the molecular studies of this important parasite in the future.

## Author summary

Schistosomiasis is an acute and chronic disease that remains one of the most prevalent and serious of the parasitic diseases in the world. Three major *Schistosoma* species cause human schistosomiasis, including *Schistosoma japonicum*, *S*. *mansoni* and *S*. *haematobium*. However, the three schistosome references or draft genomes were released in the last decade, which greatly facilitate the progress in the whole research field of schistosome. However, limited by the sequencing technique and mixture samples at that time, only a genome draft was suppled to *S*. *japonicum*, which is fragmented and difficult to be a reference in functional genome analysis and gene discovery. Here, using the combination of PacBio and Illumina Sequencing technologies, we present a high-quality assembly of *S*. *japonicum* with contig and scaffold N50 length of 871.9 kb and 1.09 Mb, representing 142.4-fold and 6.2-fold improvement over the released WGS-based assembly, respectively. The assembly genome with high quality will certainly supply a new reference genome of *S*. *japonicum* and be beneficial to functional genomic and comparative genomics of schistosome, as well as other helminths.

## Introduction

Schistosomiasis is an acute and chronic disease that remains one of the most prevalent and serious of the parasitic diseases [1]. Globally, an estimated 700 million people are at risk of infection and more than 250 million people are affected in 78 countries and territories [2, 3]. Three major *Schistosoma* species cause human schistosomiasis, including *Schistosoma japonicum*, *S*. *mansoni* and *S*. *haematobium*. *S*. *japonicum* is mainly epidemic in South China, Indonesia and the Philippines [4, 5], with 147,642 patients being treated in China in 2016 [6]. *S*. *japonicum* has a complex life-cycle that involves an aquatic snail (*Oncomelania hupensis*) as an intermediate host [7] and a wide definitive host range, infecting humans as well as more than 40 other mammals, and these make it difficult to control [8–10]. Moreover, *S*. *japonicum* is the most pathogenic among human *Schistosoma* due to a high fecundity. Each pairing of *S*. *japonicum* deposits up to 3,000 eggs per day, which is 10-fold greater than both *S*. *mansoni* or *S*. *haematobium* [11, 12]. Despite the remarkable success of schistosomiasis control over the past 60 years, this disease remains a major public health problem in South China, Indonesia and the Philippines [4, 13].

Three *Schistosoma* draft genomes (*S*. *mansoni*, *S*. *haematobium*, *S*. *japonicum*) have been published. The genome of *S*. *mansoni* was first published in 2009 with the whole genome shotgun (WGS) sequencing strategy, then improved with second generation DNA, Single-molecule real time (SMRT) sequencing technology and genetic markers. The latest genome V7 is 409.6 Mb with a N50 scaffold length of 50.5 Mb, possessing 95.9% of the genome assembled into chromosomes [14–16]. The genome of *S*. *haematobium* was assembled using Illumina-based technology, achieving an assembly of 385 Mb (29,834 scaffolds; N50 scaffold size of 317 kb) [17]. The genome draft of *S*. *japonicum* was generated by WGS sequencing strategy with mixed and outbred adult worms. This assembly was highly fragmented, including 25,048 scaffolds and 95,269 contigs with contig and scaffold N50 length of 6.12 kb and 176.9 kb [18]. Therefore, it is difficult to serve as the reference genome for curated gene prediction, as well as comparative and functional genomic analysis. An improved high-quality genome assembly of *S*. *japonicum* is urgently required.

SMRT sequencing technology from Pacific Biosciences (PacBio) can provide an opportunity to significantly improve genome assembly [19–21]. In the present study, we utilized PacBio and Illumina sequencing data from clonal worms to generate the genome of *S*. *japonicum*. Then, we re-annotated the improved genome assembly by additionally applying RNA sequencing data and performed comparative genomic analysis for *S*. *japonicum* and other six flatworms. Our improved assembly and annotation will provide valuable genomic resource for future studies, especially functional genomic analysis for *S*. *japonicum*.

## Materials and methods

### Ethics statement

All protocols involving animals were performed based on the guidelines of the Association for Assessment and Accreditation of Laboratory Animal Care International. The study procedures followed institutional ethical guidelines that were approved by the ethics committee at the National Institute of Parasitic Diseases, Chinese Center for Disease Control and Prevention (NIPD, China CDC; Permit No: IPD2008-4).

### Sample collection and sequencing

*Oncomelania hupensis* snails were individually exposed to a single miracidia of *S*. *japonicum* in tissue culture plates for 2 hours. After group cultivating for 3 months in small plastic aquaria at 25°C, each snail was checked individually for cercarial release under a strong light. Those releasing cercariae were housed subsequently in single centrifuge tube of pond water marked as “clonal”. The “clonal” cercariae from each single miracidium infection of *O*. *hupensis* snail were then used to infect Kunming mice, using 100–150 cercariae per mouse. After 30 days, 100–120 adult male worms and 60–80 adult female worms were collected by perfusing the hepatic portal system and mesenteric veins for genome sequencing, respectively. Genomic DNA (gDNA) extraction were carried out using the DNeasy Blood& Tissue Kit (Qiagen, Germany) according to manufacturer instruction, which generate 2.0 ug gDNA of male worms and 1.0 ug gDNA of female worms. The gDNA was quantified by applying Qubit dsDNA HS Assay Kit (Invitrogen, Thermo Fisher Scientific, Waltham, USA) and then by gel electrophoresis on 1% agarose gel, NanoDrop 2000/c Spectrophotometers (Thermo Fisher Scientific, Waltham, USA) and Agilent Bioanalyzer 2100 (Agilent Technologies, Santa Clara, CA).

Three 20-kb SMRTbell libraries were constructed using BluPippin Size Selection System protocol and sequenced on PacBio RS II platform using P5-C3 chemistry. Two male libraries (two clones) were sequenced in 12 SMRT cells while one female library (one clone) was sequenced in 2 SMRT cells. An Illumina paired-end library with 350 base pair (bp) insert size (PE350) was also constructed using the male gDNA and sequenced on Illumina platform for genome size estimation, correction of genome assembly and assembly evaluation.

RNA was extracted from three developmental stages of *S*. *japonicum* (i.e. adult, sporocyst and cercariae) using RNAiso Plus, respectively. Three paired-end (PE) RNA-seq libraries were generated using TruSeq RNA Library Preparation Kit v2 with different insert sizes (150 bp for adult and 250 bp for sporocyst and cercariae) and then sequenced on Illumina platform.

### Genome estimation and assembly

Pacbio sequencing reads were analyzed for sequence quality with FastQC v0.11.8 [22]. The adaptor contamination, PCR duplication, low-quality reads and mice contamination of all Illumina sequencing data were filtered out using Trimmomatic v0.36 [23] before genome estimation and assembly. To estimate genome of *S*. *japonicum*, jellyfish v1.1.12 was used to construct a k-mer frequency spectrum (k = 21) with PE350 cleaned sequencing data, and GenomeScope [24] were then performed to estimate the genome based on 21-kmer frequency.

The *de novo* genome assembly was executed by wtdbg v1.1.006 [25]. The WTDBG assembled raw reads without error correction and built the consensus from intermediate assembly output. Therefore, wtdbg-cns, minimap [26] and map2dbgcns in WTDBG were applied for initial error-correction process. Further, we polished the consensus genome assembly using pbalign v0.3.1 and Arrow v2.2.1 (Pacific Biosciences) with SMRT long reads, followed by polishing with pilon v1.22 [27] using PE350 clean sequencing reads. Finally, P_RNA_scaffolder [28] was used for scaffolding genomes with 150-bp library RNA-seq reads. Assembly statistics of final assembled genome (V2) were assessed with QUAST v4.6.3 [29].

### Genome evaluation

To evaluate the completeness and accuracy of the V2 assembly of *S*. *japonicum*, we mapped PE350 clean data to both the V2 assembly and the first genome (v1) of *S*. *japonicum* [18] using bwa v0.7.12 [30]. Average sequencing depth and mapping rates were calculated using SAMTOOLS v1.8 [31]. The completeness of the coding gene sets was also evaluated by benchmarking universal single-copy orthologous genes (BUSCO v3) [32]. Last, a synteny analysis was also performed using SyMAP v4.2 [33] to further assess the quality of V2 assembly, considering only scaffolds of at least 100 kb and ordering the *S*. *japonicum* scaffolds based on chromosome-level *Schistosoma mansoni* genome V7 [14, 15] form WormBase ParaSite database [34]. We determined synteny for *S*. *mansoni* against both the v1 and v2 assembly for comparison. The genomes of *S*. *mansoni*, *S*. *haematobium* and other organisms for comparison were retrieved in WormBase Parasite database (S1 Table) [34].

### Protein coding gene prediction and ncRNA prediction

By applying RepeatMasker v4.0.7 [35] with Repbase [36]and a *de novo* repeat database constructed with RepeatModeler v1.0.11 [37], the repeats elements were identified in V2 assembly before protein-coding gene prediction. Gene prediction was conducted by combining *ab initio* prediction, homology-based prediction and transcriptome-based prediction. AUGUSTUS v2.5.5 [38], SNAP v2006-07-28 [39] and GeneMark-ES v4.33 [40] with default parameters were used for the *ab initio* gene prediction in the repeat-masked genome. GeMoMa v2.3 were applied for the homology-based gene prediction, and the protein repertoires of flatworms including *S*. *japonicum* (GCA_000151775.1) [18], *S*. *mansoni* (GCA_000237925.2) [14, 15], *S*. *haematobium* (GCA_000699445.1) [17], *Clonorchis sinensis* (GCA_000236345.1) [41], *Opisthorchis viverrini* (GCF_000715545.1) [42], *Hymenolepis microstom* (GCA_000469805.2) [43], *Echinococcus multilocularis* (GCA_000469725.3) [43], *Echinococcus granulosus* (GCA_000524195.1) [44] from GenBank were used as the references. For the transcriptome-based gene prediction, All RNA-seq data were assembled by Trinity v2.7.0 [45]. The Trinity assembly contain 270,293 transcripts with N50 length of 1,155 bp. These assembled sequences plus 106,621 expressed sequence tags (EST) and mRNA from GenBank were aligned against the V2 assembly by Program to Assemble Spliced Alignment (PASA) [46, 47]. Valid transcript alignments were clustered based on genome mapping location and assembled into gene models. Besides, RNA-seq reads were also directly mapped to the genome by HISAT2 v2.1.0 [48] and assemble into gene models by StringTie v1.3.4 [49]. Gene models generated from all the above methods were integrated by EvidenceModeler (EVM) [49]. The gene models were further updated to generate untranslated regions (UTRs) and alternative splicing variation by PASA [50, 51]. Finally, limit manual refinement of genome annotations was perform using Apollo [52] to fix reading frames. Then, the predicted genes length were summarized with an R package “GenomicFeatures” [53].

In addition, the non-coding RNAs (ncRNAs) including miRNA, rRNA, snRNA, and tRNA were also predicted. tRNAs were annotated by tRNAscan-SE v1.3.1 with default parameters for eukaryotes [54], snRNAs were extracted by INFERNAL v1.1.2 [55] against the RFAM v14.0 database [56], rRNAs were predicted by RNAmmer v1.2 [57], miRNA were annotated with deep-sequencing data from Sequence Read Archive (SRA) (SRR2927289) using miRDeep2 [58].

Gene functions of protein-coding genes were annotated by a successive blastp v2.7.1 [59] analysis against Swiss-Prot [60], TrEMBL and non-redundant (NR) databases [61] with an *E*-value cut-off of 10^−5^. Gene domains were annotated using InterProScan5 [62] and HMMER v3.2.1 [63] against the PFAM database [64]. Gene Ontology (GO) [65, 66] annotations were generated combining the results from InterProScan5 and blastp analysis against NR database using BLAST2GO_CLI v1.15 [67]. Additional functional information was also derived via pathway analysis based on homology to the characterized pathways in Kyoto Encyclopaedia of Genes and Genomes (KEGG) [68] with online KAAS [69].

### Gene family identification and phylogenetic analysis

To investigate gene family evolution in the *S*. *japonicum* genome, nucleotide and protein sequence of *S*. *mansoni*, *S*. *haematobium*, *C*. *sinensis*, *O*. *viverrini*, *Fasciola hepatica* and *H*. *diminuta* were retrieved from WormBase ParaSite databases [34]. Only the proteins from the longest transcripts were retained for each gene locus with alternative splicing variants. Those proteins with length ≥ 20 aa were used to calculate pair-wise similarities all-against-all BLASTp with *E*-value cut-off of 1e^-10^, and low-quality hits (coverage < 50%) were removed. Orthologous groups were further constructed by OrthoMCL v2.0.9 [70] with an inflation parameter of 1.5.

A total of 2,322 Single copy genes were retrieved for divergence time estimation. Proteins from each single-copy families were aligned using MAFFT v7.407 [71] with the parameter of “—localpair—maxiterate 1000”. The corresponding CDS alignments were back-translated from the corresponding protein alignments with pal2nal, followed by the removal of poorly aligned regions using trimAl v1.2 with automated parameter [72]. The curated alignments of each family were concatenated into a super alignment matrix using phyutility [73]. Divergence times were estimated from a refined concatenated CDS alignment using BEAST2 v2.5.1 with a strict clock model. Priors used calibrated Yule model, time calibration constrains with two previously estimated dates between Trematoda and Cestoda (~106 million years ago (Mya)) and between Opistorchiida and Schistosomatoidea (~70 Mya) [74], and default setting for other priors. Samples from the posterior were drawn every 1,000 steps over a total of 10,000,000 steps per MCMC run.

### Gene family evolution

Gene family expansion and contraction analyses was performed with Computational Analysis of gene Family Evolution software (CAFÉ v4.0.1) [75] according to divergence times. For each gene family, CAFÉ generated a family-wide *P* value and A branch/node-specific Viterbi *P* value indicating a possible gene-family expansion or contraction event. In this study, family-wide *P*-value less than 0.05 and a branch/node ‘Viterbi *P*-value’ less than 0.001 was considered as a signature of significant expansion or contraction for a specific gene family and specific species, respectively. Enrichment of GO terms for unique and expanded gene families of *S*. *japonicum* were identified using clusterProfiler package [76] using the Benjamini-Hochberg FDR correction. Significantly enriched GO terms were identified with corrected *P* value of < 0.05. KEGG pathway enrichment were performed with KOBAS v3.0 software [77, 78].

### Accession numbers

The raw sequencing data are available via NCBI under SRA accessions PRJNA515567. This Whole Genome Shotgun project has been deposited at DDBJ/ENA/GenBank under the accession SKCS00000000. The version described in this paper is version SKCS01000000.

## Results

### Genome assembly

**Fig 1:**
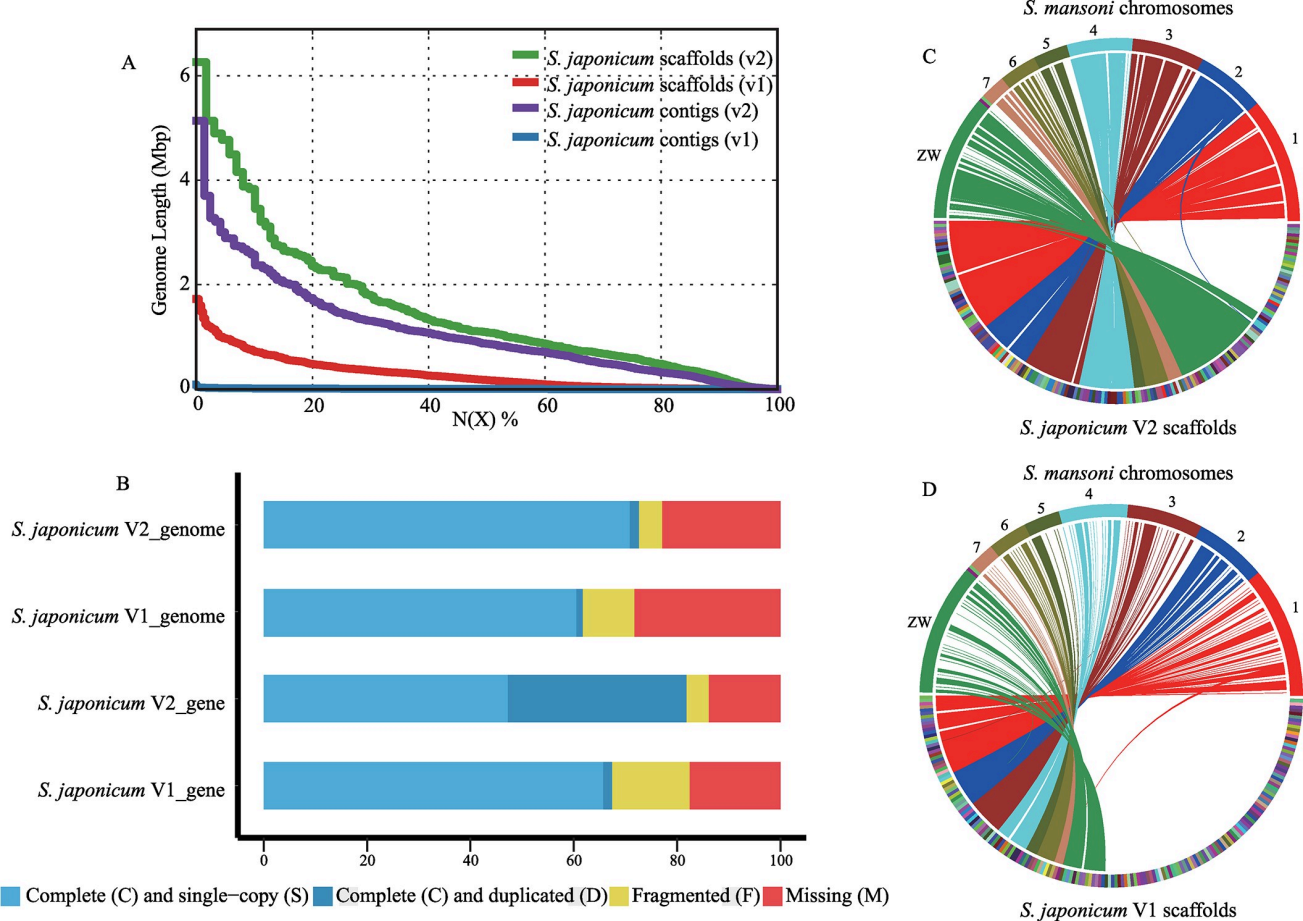
Genome assembly of *Schistosoma japonicum*. (A) Comparison of contiguity between the two versions of *S*. *japonicum* genome assembly. N(x)% graph shows the contig and scaffold sizes (y-axis), where x% of the genome assembly consists of contigs and scaffolds of at least that size. (B) comparison between two version of *S*. *japonicum* genome assembly, showing the portions of the genomes that are complete (blue), fragmented (yellow) or missing (red), as determined by benchmarking universal single-copy orthologs (BUSCO) analysis with metazoan_odb9 database. (C) Circle plot of synteny between the second version of *S*. *japonicum* genome and *S*. *mansoni* genome V7 made using SyMAP. It shows a high degree of synteny, with many long *S*. *japonicum* scaffolds covering significant portions of *S*. *mansoni* chromosome. (D) Circle plot of synteny between the first version of *S*. *japonicum* genome and *S*. *mansoni* genome. V1 indicated conventional capillary sequenced genome and V2 indicated our improved genome.

A total of 20 G PacBio sequences, which has an average length of 7.4 kb and average sequence quality (Phred score) of 10 (S1 Fig), and 80 G Illumina paired-end (PE) clean sequences were generated from the whole genome sequencing. Additionally, a total of 33 G PE clean sequences was also generated for RNA samples on the Illumina platforms (S2 Table). The estimated genome size was approximately 375,706,643 bp based on 21-kmer analysis from the PE350 library, with the main peak at a depth of approximately 157× (S2 Fig). In the 21-kmer frequency distribution, the second peak at approximately half the coverage value of the main peak, indicates the high heterozygosity (1.05%) of the *S*. *japonicum* genome. The final assembled *S*. *japonicum* genome had a total length of 369.9 Mb (98.5% of the estimated genome) with 1,789 scaffolds and 33.76% GC ratio (S3 Fig). The overall assembly statistics of the improved genome version (*S*. *japonicum* V2) were dramatically improved when compared with the previously released version (*S*. *japonicum* V1; S1 Table) [18]: contig N50 increased from 6.12 kb to 871.9 kb, the scaffold N50 increased from 176 kb to 1.09 Mb and the number of gaps decreased from 70,219 to 319 (Table 1, Fig 1A).

**Table 1:** Genome assembly statistics for the improved genome of *S*. *japonicum* in comparison with three published *Schistosoma* genome. V1 indicated conventional capillary sequenced genome and V2 indicated our improved genome.

	*S*. *japonicum* V2	*S*. *japonicum* V1	*S*. *mansoni*	*S*. *haematobium*
Genome size (bp)	369,900,518	402,743,189	409,579,008	375,894,156
Number of scaffolds	1,789	25,048	320	29,834
Number of contigs	2,108	95,267	602	59,195
Longest scaffold (bp)	6,264,197	1,730,213	88,881,357	1,826,302
Average scaffold length (bp)	210,145	16,078	1,279,934	12,560
Number of scaffolds: >10 kb	1,052	4,707	318	2,384
Number of Gaps	319	70,219	282	29,361
Scaffold N50 (bp)	1,093,989	176,869	50,458,499	317,484
Contig N50 (bp)	871,911	6,121	5,339,380	22,446
GC content (%)	33.76	34.08	35.47	34.22
Repeat content (%)	46.87	44.56	49.23	42.83

### Genome quality evaluation

More than 90.3% of the high-quality PE350 reads could be mapped concordantly to the V2 assembly, which is an improvement when compared with those of the V1 assembly (S3 Table). Besides, approximately 95.7% of the V2 assembly had a sequencing depth > 10-fold (S4 Fig) indicating high accuracy at the nucleotide level of V2 assembly. Assessment of genome completeness using BUSCO analysis confirmed that 85.2% of the 303 core eukaryotic genes and 72.6% of the 978 core metazoan genes were completely presented in the V2 assembly (Fig 1B). The BUSCO results were similar with those of recent studies for other flatworms and represent an improvement over the V1 assembly (S4 Table). Synteny were computed between *S*. *japonicum* and the chromosome-level *S*. *mansoni* genomes V7 and both the V2 and V1 assemblies were used for comparison. The increased contiguity of V2 assembly allowed us to compute synteny between *S*. *japonicum* and *S*. *mansoni* genome, more than doubling the percentage of the genome covered by synteny blocks from 29% to 67% and increasing the size of synteny blocks, with 97 of 277 synteny blocks > 1 Mb in length (Fig 1C and 1D).

### Gene annotation

**Fig 2:**
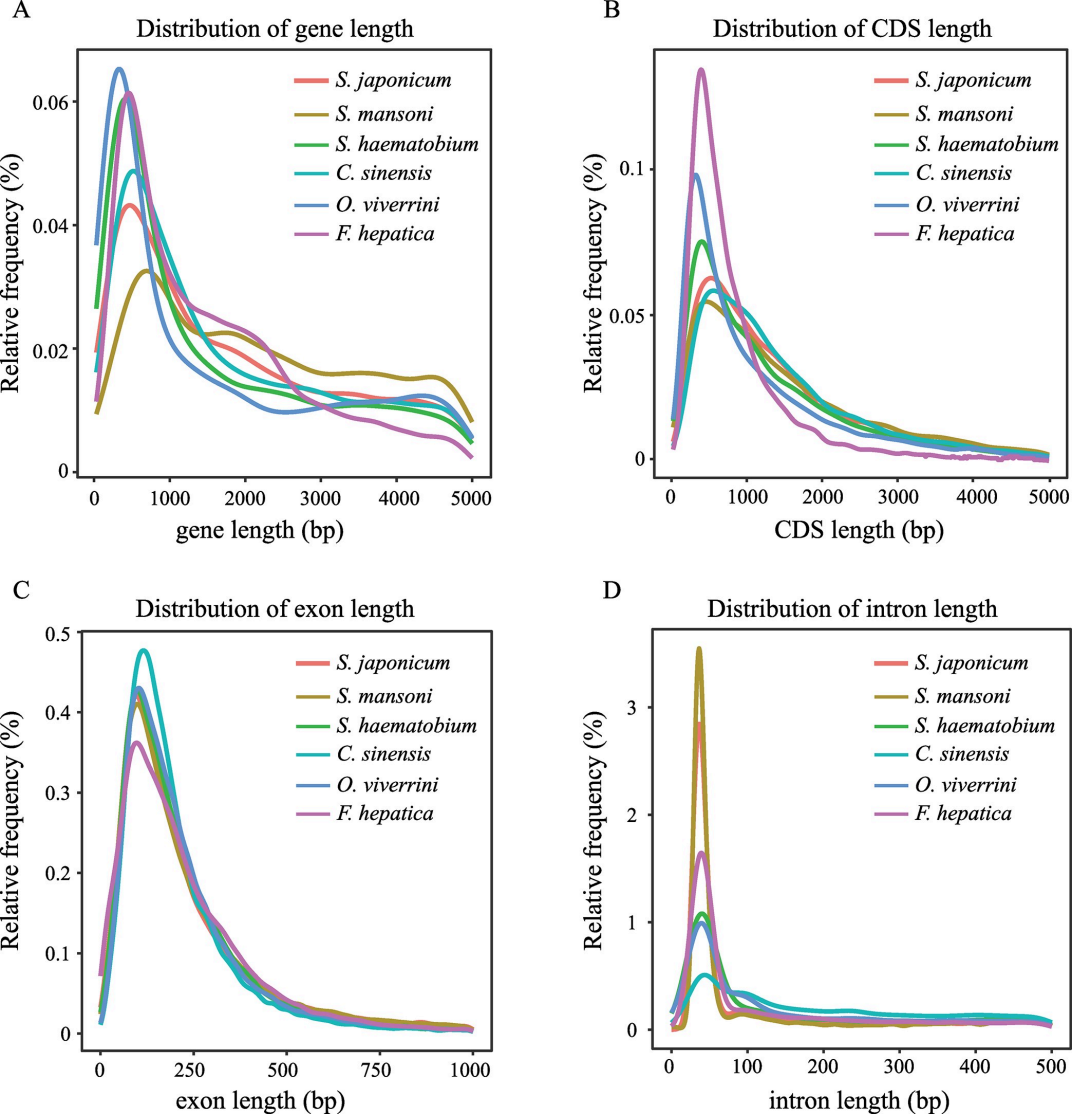
Length distribution comparison on total gene, CDS, exon, and intron of annotated gene models of the *S*. *japonicum* with other closely related Trematoda species. Length distribution of total genes (A), CDS (B), exon (C), and intron (D) were compared to those of *S*. *mansoni*, *S*. *haematobium*, *C*. *sinensis*, *O*. *viverrini*, and *F*. *hepatica*.

Approximately 46.87% of V2 assembly was identified as repeat elements, which is similar with the V1 assembly (44.56%) and the close relatives *S*. *mansoni* (49.23%) and *S*. *haematobium* (42.83%). Long interspersed elements were the most predominant elements, which account for 19.89% in V2 assembly (S5 Table). By combining *de novo* prediction, homology-based prediction and transcriptome-based prediction, a total of 10,089 protein-coding genes and 16,936 transcripts were predicted in the V2 assembly. Of the 10,089 protein-coding genes in the V2 assembly, 9,387 (93.0%) were supported by RNA-Seq clean data and 1,207 were newly detected genes. The number of predicted genes in V2 assembly is significantly lower (79.2%) than those in V1 assembly. Furthermore, BUSCO analysis with the metazoan_odb9 database [32] showed that the proportion of complete genes increased from 67.4% in the V1 assembly to 81.8% in our V2 assembly, while the ratio of the fragmented genes decreased from 15% to 4.3% (Fig 1B, Table 2). These results indicated that the large number of contigs in V1 assembly comprised a substantial number of fragmented or misassembled sequences, resulting in an overestimate of the number of unique protein-coding genes. The average gene length and average coding DNA sequence (CDS) size were 18,370 bp and 1,537 bp, which were longer than those in V1 gene annotation (Table 2). Additionally, the gene number, gene length distribution, CDS length distribution, exon length distribution and intron length distribution were similar with those in other Trematoda species (Fig 2). Among the 10,089 predicted genes, 9,291 (92.1%) genes had matches in the NR database, 6,642 (65.8%) generated hits to Swiss-Prot database, 8,689 (86.1%) were identified in InterPro, 8,368 (82.9%) and 4,547 (45.1%) were assigned GO terms and KEGG pathways (Table 3, S6 Table). Besides, four types of non-coding RNAs were also identified, including 172 miRNAs, 1,263 tRNAs, 10 rRNAs and 54 snRNAs (Table 3).

**Table 2:** Comparison of predicted genes of the two version of *S*. *japonicum* genome assembly. V1 indicated conventional capillary sequenced genome and V2 indicated our improved genome.

	*S*. *japonicum* (V2)	*S*. *japonicum* (V1)
Gene number	10,089	12,738
Average gene length (bp)	18,370	9,960
Average CDS length (bp)	1,537	1,172
Average exons per gene	8.3	5.3
Average exon length (bp)	370	223
Average intron length (bp)	2,521	2,058
**BUSCO analysis**		
Complete	81.8%	67.4%
partial	4.3%	15.0%
Missing	13.9%	17.6%

**Table 3:** Annotation of protein-coding genes and noncoding RNA elements in the improved *S*. *japonicum* genome assembly.

	Number (%)
**Protein annotations**	
SWISSPROT	6,642 (65.8)
TrEMBL	6,137 (60.8)
NCBI nr database	9,291 (92.1)
KEGG database	4,547 (45.1)
InterProScan	8,689 (86.1)
Gene ontology annotation	8,368 (82.9)
**Conserved noncoding RNA elements**	
Small nuclear RNA (snRNA)	54
Transfer RNA (tRNA)	1,263
Micro RNA (miRNA)	172
Ribosomal RNA (rRNA)	10

### Genome family evolution

**Fig 3:**
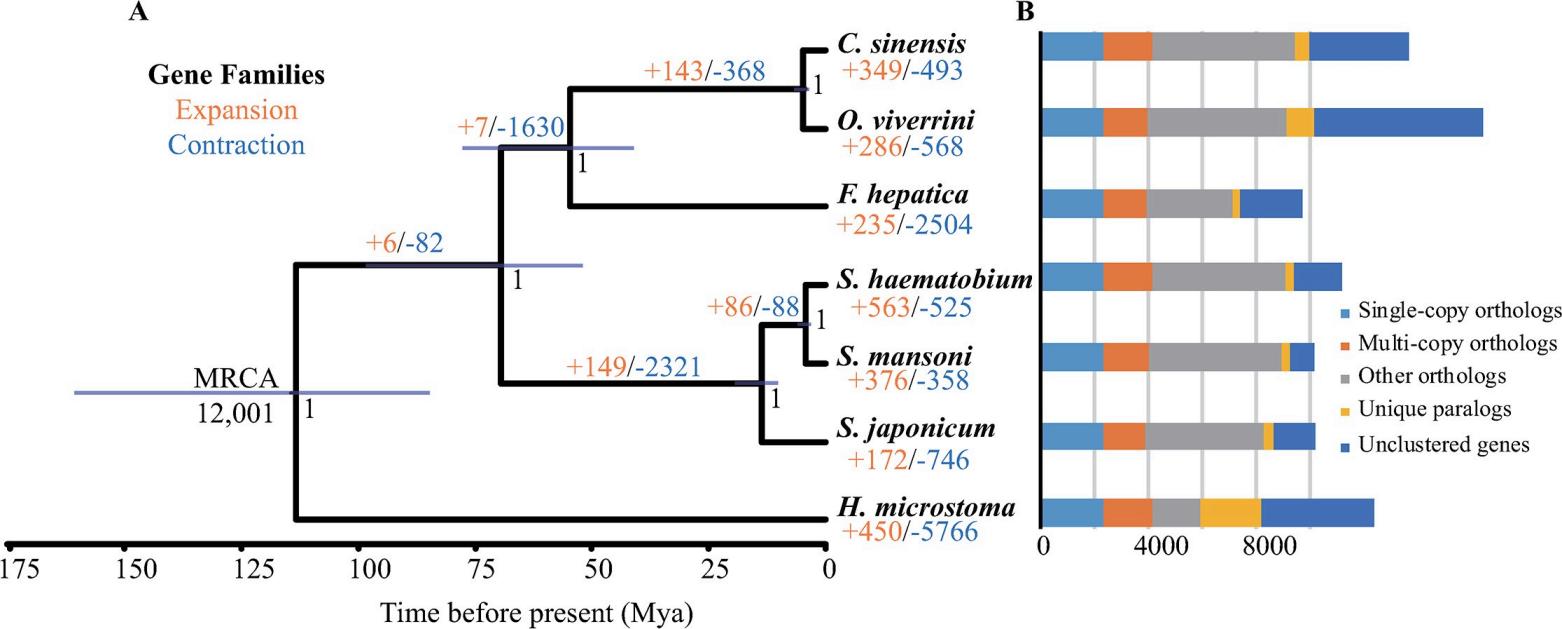
Comparative genome analysis between *S*. *japonicum* and other six flatworms. (A) Phylogenetic tree and expansion and contraction of gene families. The phylogenetic tree and divergence time were generated from 2,322 single-copy orthologous genes using BEAST2. The branch lengths of the phylogenetic tree are scaled to estimated divergence time. Tree topology is supported by posterior probability of 1.0 for all nodes. The blue bars on the nodes indicate the 95% credibility intervals of the estimated posterior distributions of the divergence times. The number of expanded (orange) and contracted (blue) gene families is designated on each branch. Bar charts indicates the orthologous and paralogous gene families in *S*. *japonicum* and other six flatworm species. (B) Comparison of the number of gene families in 7 Platyhelminthes species.

Comparative analysis between *S*. *japonicum* and other six Platyhelminthes species was conducted. In brief, 12,001 gene families were constructed in all 7 species using OrthoMCL [70], 2,322 gene families were identified as single-copy orthologous gene families, and 3,798 gene families were common to all 7 species. Furthermore, 8,278 orthologous genes were detected in *S*. *japonicum* genome, and 103 gene families corresponding 351 genes that were specific to *S*. *japonicum* (Fig 3B, S7 Table). These species-specific genes were significantly enriched in molecular functional categories related to RNA-directed DNA polymerase activity, endonuclease activity, nucleic acid binding, ribonuclease T2 activity and metalloendopeptidase activity (S8 Table).

**Fig 4:**
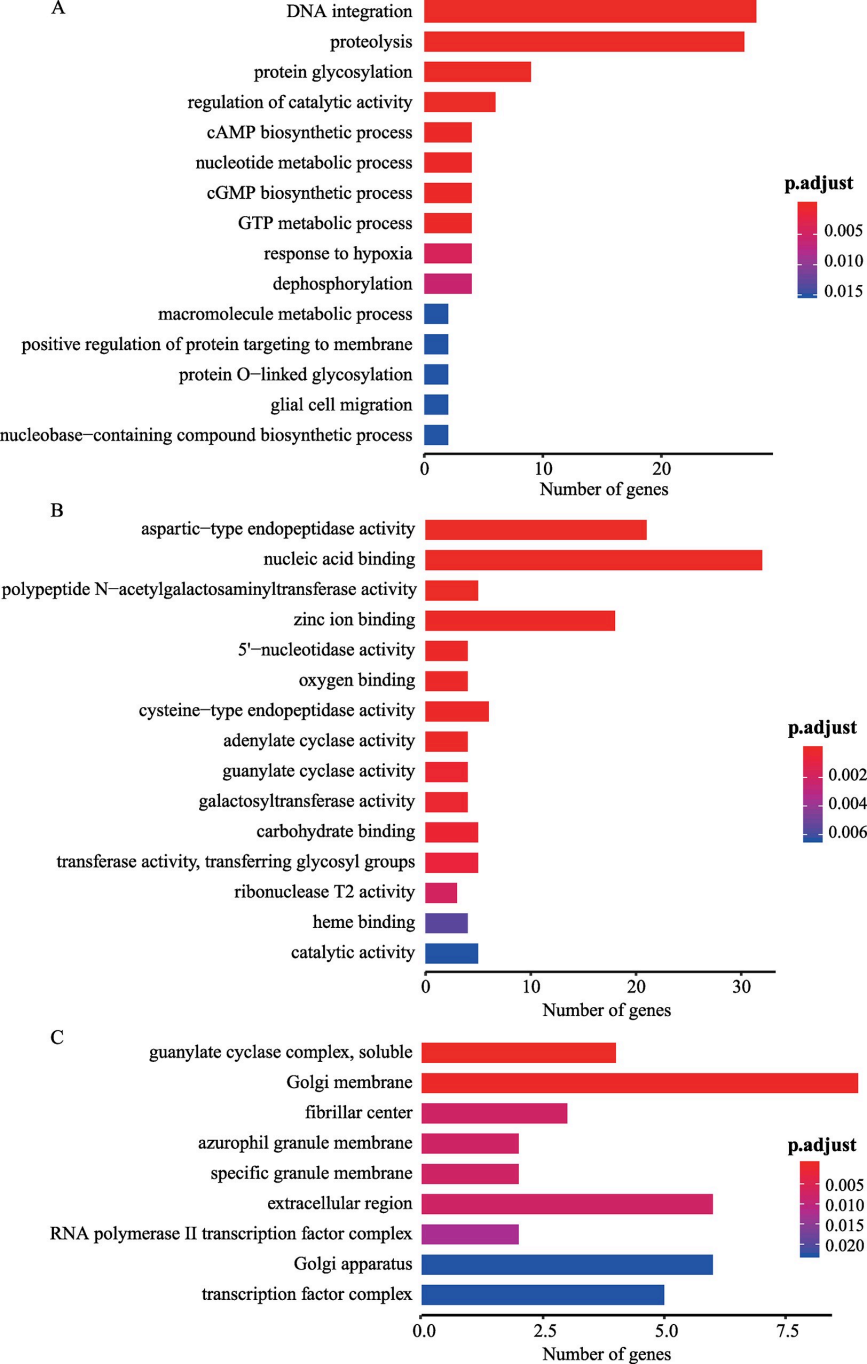
Gene Ontology enrichment analysis of significantly expanded gene families. (A) biological processes, (B) molecular function and (C) cellular component.

The phylogenetic analysis based on 2,322 single-copy orthologs showed that the divergence time of *S*. *japonicum*-*S*. *mansoni* and *S*. *mansoni*-*S*. *haematobium* occurred at ~14 Mya and ~4 Mya (Fig 3A), which are consistent with previous estimates [79, 80]. 20 significantly expanded and 5 significantly contracted gene families were identified in *S*. *japonicum* (S9 Table). The genes from these significantly expanded families were mainly enriched in nucleic acid binding, polypeptide N-acetylgalactosaminyltransferese activity, 5'-nucleotidase activity, cysteine-type endopeptidase activity, galactosyltransferase activity, adenylate cyclase activity and ribonuclease T2 activity (Fig 4, S10 Table). In addition, these expanded genes were also significantly enriched in the KEGG pathway related to mucin type O-Glycan biosynthesis (Corrected *P* value = 2.77e-06).

## Discussion

A draft genome of *S*. *japonicum* was released in 2009 and provided a resource for gene discovery and data mining. However, the genome was generated using WGS sequencing strategy with mixed and outbred adult worms, resulting its highly fragments in the assembly. Here, by applying combination of the long-reads PacBio and short-reads Illumina sequencing data from the clonal worms, we provided an improved high-quality genome assembly (V2) of *S*. *japonicum*. When compared to the released WGS genome (V1) of *S*. *japonicum* [18], our V2 assembly showed significant improvements in assembly contiguity, accuracy and completeness, with 142.4-fold increase in contig N50, 6.2-fold increase in scaffold N50, 5% improvement in the mapping rate of PE reads and 10% improvement in the proportion of complete genes. Genome annotation has also upgraded by using deep coverage transcriptome data. Over 93.0% of the 10,089 protein-coding genes are supported by RNA-Seq data and 1,207 new genes were predicted in our improved assembly. Also, the length of protein-coding genes and completeness were increased significantly, when compared with those of the V1 assembly. Here, we found that genes typically had large introns (the average length of 2,521 bp) and much smaller exons (the average length of 370 bp). This pattern was also detected in *S*. *mansoni* [14] and in many other eukaryotes [81]. The large introns and much smaller introns might be attributable to the high activity of transposable elements [81].

Applying our improved V2 assembly, 20 significantly expanded gene families in *S*. *japonicum* were detected. Specifically, expansion of cathepsin B-like cysteine proteinase genes in *S*. *japonicum* could be associated with host invasion, hemoglobin degradation and immune invasion [82]. Another expanded gene family encoding Trematoda eggshell synthesis protein could compose the surface of the eggs, and thus protect the embryo from environmental challenges [83]. Previous study has shown that this family is essential for vitellarium development and egg production in *S*. *japonicum* [84]. Additionally, we detected expansions in the gene families that encode ribonuclease Oy, polypeptide N-acetylgalactosaminyltransferase (ppGalNAcTs) and N-acetylglucosaminyltransferase (UDP-GlcNAc). Ribonuclease Oy is an egg antigen molecule and induce a polarized Th2 type host immune response, which may enable eggs to escape from host tissues and initiate granuloma formation [85]. ppGalNAcTs and UDP-GlcNAc are important in the biosynthesis of glycan and glycoconjugates that interact with both the innate and adaptive arms of immunity in human and animal hosts [86, 87]. We detected 103 gene families (351 genes) that were specific to *S*. *japonicum*. Interestingly, they mainly related to the central metabolism, such as RNA-directed DNA polymerase activity and proteolysis. We assumed that the function of “proteolysis” was related with invasion and hemoglobin degradation of *S*. *japonicum* [88, 89]. Therefore, this function might lead to the wide host range of *S*. *japonicum*, when compared with other *Schistosoma* species [8]. The pao retrotransposon and reverse transcriptase, which was related with RNA-directed DNA polymerase activity, were regarded as principal forces driving the evolution of eukaryotic genomes [90]. Moreover, the species-specific duplication of the retrotransposon and reverse transcriptase were also observed in *S*. *haematobium* and *S*. *mansoni*, which imply that these genes could drive the genome divergence. Additionally, we found 32 egg proteins that were specific to *S*. *japonicum*; these proteins might be related with the egg formation and thus lead to a higher fertility, when compared to *S*. *mansoni* or *S*. *haematobium* [12]. These expanded and unique genes could be potential targets to investigate the molecular mechanisms of adaptations to diverse definitive host and high egg production of *S*. *japonicum*, and thus will provide the candidates for vaccine and drug targets.

Overall, our improved genome assembly of *S*. *japonicum*, together with its newly annotation, will serves as a framework for the functional analysis of *S*. *japonicum*, which may facilitate the development of new disease interventions for its control and eventual elimination.

## Supporting information

S1 Fig Distribution of quality score over all PacBio sequences. (EPS)

S2 Fig Graph of K-mer distribution (K = 21) generated from the PE350 library using GenomeScope. The big peak at the coverage of 157 in the graph is the homozygous portion of the genome, which accounts for the strands of the DNA having identical 21-kmers. The smaller shoulder to the left of the peak corresponds to the heterozygous portion of the genome, which accounts for the strands of the DNA having different 21-kmers. The *S*. *japonicum* genome size was estimated to be 375.7 Mb.
(EPS)

S3 Fig The GC distribution of the new version of *S*. *japonicum* genome assembly. (EPS)

S4 Fig Sequencing depth distribution of the new version of *S*. *japonicum* genome assembly. (EPS)

S5 Fig Comparison of genome completeness between the genome of *S*. *japonicum* and other Trematoda species based on BUSCO evaluation, using either the eukaryote_odb9 database (A) and metazoa_odb9 database (B). The portions of the genomes that are complete (blue), fragmented (yellow), or missed (red), are determined by benchmarking universal single-copy orthologs (BUSCO) analysis for eukaryotic-conserved (left) and metazoan-conserved (right) genes.
(EPS)

S1 Table Species used for comparison of genome parameters and genomic comparative analysis. (DOCX)

S2 Table Summary of improved genome sequencing data generated using PacBio and Illumina platform. (DOCX)

S3 Table Comparison of mapping rates for PE350 between the two versions of the *S*. *japonicum* genome assembly. V1 indicated conventional capillary sequenced genome and V2 indicated our improved genome.
(DOCX)

S4 Table BUSCO results for completeness assessment for the first, second version assemblies of *S*. *japonicum* and genome of other Trematoda species, using either the eukaryote or metazoan databases. (XLSX)

S5 Table Composition of repetitive elements in the two version of *S*. *japonicum* genome assembly. V1 indicated conventional capillary sequenced genome and V2 indicated our improved genome.
(DOCX)

S6 Table Annotations of the predicted gene models. (XLSX)

S7 Table Unique gene families in *Schistosoma japonicum* based on OrthoMCL analysis. (XLSX)

S8 Table Gene ontology (GO) enrichment analysis for unique gene families of *Schistosoma japonicum*. (MF: molecular function; CC: cell component; BP: biological process).
(DOCX)

S9 Table Gene families that showed significant expansions in *Schistosoma japonicum* based on CAFE4 analysis. (sja: *S*. *japonicum*; smm: *S*. *mansoni*; shx: *S*. *haematobium*; ovi: *O*. *viverrini*; csi: *C*. *sinensis*; fhe: *F*. *hepatica*; hmi: *H*. *microstoma*).
(XLSX)

S10 Table Gene ontology (GO) enrichment analysis for expanded gene families of *S*. *japonicum*. (DOCX)
