# Supplementary material for: An improved genome assembly of the fluke Schistosoma japonicum
Source: PLoS Negl Trop Dis. 2019 Aug 7;13(8):e0007612. doi: 10.1371/journal.pntd.0007612 (PMC6685614; doi:10.1371/journal.pntd.0007612)
Supplement: S1 Table — (DOCX) [file pntd.0007612.s006.docx]

S1 Table. Species used for comparison of genome parameters and genomic comparative analysis.

| **Species name** | **Category** | **URLs** |
| --- | --- | --- |
| *Schistosoma japonicum v1* | Trematoda | https://parasite.wormbase.org/Schistosoma_japonicum_prjea34885/Info/Index/ |
| *Schistosoma mansoni* | Trematoda | https://parasite.wormbase.org/Schistosoma_mansoni_prjea36577/Info/Index/ |
| *Schistosoma haematobium* | Trematoda | https://parasite.wormbase.org/Schistosoma_haematobium_prjna78265/Info/Index/ |
| *Opisthorchis viverrini* | Trematoda | https://parasite.wormbase.org/Opisthorchis_viverrini_prjna222628/Info/Index/ |
| *Clonorchis sinensis* | Trematoda | https://parasite.wormbase.org/Clonorchis_sinensis_prjda72781/Info/Index/ |
| *Fasciola hepatica* | Trematoda | https://parasite.wormbase.org/Fasciola_hepatica_prjeb25283/Info/Index/ |
| *Hymenolepis microstoma* | Cestoda | https://parasite.wormbase.org/Hymenolepis_microstoma_prjeb124/Info/Index/ |
