# Supplementary material for: An improved genome assembly of the fluke Schistosoma japonicum
Source: PLoS Negl Trop Dis. 2019 Aug 7;13(8):e0007612. doi: 10.1371/journal.pntd.0007612 (PMC6685614; doi:10.1371/journal.pntd.0007612)
Supplement: S2 Table — (DOCX) [file pntd.0007612.s007.docx]

S2 Table. Summary of improved genome sequencing data generated using PacBio and Illumina platform.

| Libraries | Insert Size (bp) | Raw data | | Cleaned reads (%) | | |
| --- | --- | --- | --- | --- | --- | --- |
|  |  | No. of reads | Total length (Gbp) | No. of reads | Total length (Gbp) | Coverages^a^ |
| PacBio | - | 2,720,690 | 20.05 | - | - | 53.3 |
| PE350 | 350 | 569,398,602 | 85.41 | 541,664,054 (95.1%) | 80.22 (93.9%) | 213.5 |
| Adult RNA-Seq | 150 | 220,587,694 | 22.28 | 191,887,142 (87.0%) | 18.99  (85.2%) | 50.5 |
| Cercaria RNA-Seq | 250 | 46,599,002 | 6.99 | 46,027,508 (98.8%) | 6.90 (98.8%) | 18.4 |
| Sporocyst RNA-Seq | 250 | 47,252,128 | 7.09 | 46,703,970 (98.8%) | 7.01  (98.8%) | 18.6 |

^a^Coverages is calculated with an estimate genome size of *S. japonicum* of 375.7 Mb.
