# Supplementary material for: An improved genome assembly of the fluke Schistosoma japonicum
Source: PLoS Negl Trop Dis. 2019 Aug 7;13(8):e0007612. doi: 10.1371/journal.pntd.0007612 (PMC6685614; doi:10.1371/journal.pntd.0007612)
Supplement: S3 Table — V1 indicated conventional capillary sequenced genome and V2 indicated our improved genome. (DOCX) [file pntd.0007612.s008.docx]

S3 Table. Comparison of mapping rates for PE350 between the two versions of the *S. japonicum* genome assembly. V1 indicated conventional capillary sequenced genome and V2 indicated our improved genome.

|  | Mapping rate (%) | Mapping rate (with pair) (%) ^a^ | Concordantly mapping rate (%) ^b^ |
| --- | --- | --- | --- |
| *S. japonicum* V2 | 94.2 | 93.5 | 90.3 |
| *S. japonicum* V1 | 93.1 | 91.1 | 86.7 |

^a^ Percent of reads were both pairs mapped.

^b^ Concordantly mapped: pairs mapping at the expect insert size and with the right orientation.
