# Supplementary material for: An improved genome assembly of the fluke Schistosoma japonicum
Source: PLoS Negl Trop Dis. 2019 Aug 7;13(8):e0007612. doi: 10.1371/journal.pntd.0007612 (PMC6685614; doi:10.1371/journal.pntd.0007612)
Supplement: S5 Table — V1 indicated conventional capillary sequenced genome and V2 indicated our improved genome. (DOCX) [file pntd.0007612.s010.docx]

S5 Table. Composition of repetitive elements in the two version of *S. japonicum* genome assembly. V1 indicated conventional capillary sequenced genome and V2 indicated our improved genome.

|  | V2 | | | V1 | | |
| --- | --- | --- | --- | --- | --- | --- |
| Class | Number | Length (Mbp) | Percentage | Number | Length (Mbp) | Percentage |
| LINE | 160,751 | 73.58 | 19.89 % | 161,630 | 72.37 | 17.97 % |
| LTR element | 14,829 | 12.38 | 3.35 % | 22,875 | 15.86 | 3.94 % |
| DNA element | 7,146 | 2.83 | 0.77% | 8,522 | 2.79 | 0.69 % |
| Simple repeat | 72,132 | 6.04 | 1.63 % | 70,265 | 3.69 | 0.92 % |
| Unclassified | 365,148 | 21.31 | 19.61 % | 385,369 | 84,.74 | 21.04 % |
| Low complexity | 6,459 | 0.31 | 0.08% | 5,835 | 0.27 | 0.07 |
| Total | 622,373 | 173.40 | 46.87 % | 648,661 | 179.46 | 44.56% |
