# Supplementary material for: An improved genome assembly of the fluke Schistosoma japonicum
Source: PLoS Negl Trop Dis. 2019 Aug 7;13(8):e0007612. doi: 10.1371/journal.pntd.0007612 (PMC6685614; doi:10.1371/journal.pntd.0007612)
Supplement: S8 Table — (MF: molecular function; CC: cell component; BP: biological process). (DOCX) [file pntd.0007612.s013.docx]

S8 Table. Gene ontology (GO) enrichment analysis for unique gene families of *Schistosoma japonicum*. (MF: molecular function; CC: cell component; BP: biological process).

| ID | Class | *P* value | Corrected *P* value | Count | Description |
| --- | --- | --- | --- | --- | --- |
| GO:0003964 | MF | 4.03E-64 | 2.66E-62 | 54 | RNA-directed DNA polymerase activity |
| GO:0004519 | MF | 2.42E-44 | 7.98E-43 | 39 | endonuclease activity |
| GO:0003676 | MF | 1.47E-13 | 3.22E-12 | 37 | nucleic acid binding |
| GO:0004190 | MF | 9.46E-12 | 1.56E-10 | 17 | aspartic-type endopeptidase activity |
| GO:0033897 | MF | 1.13E-09 | 1.49E-08 | 7 | ribonuclease T2 activity |
| GO:0004523 | MF | 2.14E-09 | 2.36E-08 | 8 | RNA-DNA hybrid ribonuclease activity |
| GO:0004222 | MF | 4.33E-09 | 4.08E-08 | 12 | metalloendopeptidase activity |
| GO:0003887 | MF | 1.85E-06 | 1.52E-05 | 8 | DNA-directed DNA polymerase activity |
| GO:0043167 | MF | 3.86E-06 | 2.83E-05 | 5 | ion binding |
| GO:0016301 | MF | 1.33E-05 | 8.75E-05 | 12 | kinase activity |
| GO:0097159 | MF | 3.53E-05 | 0.000212 | 5 | organic cyclic compound binding |
| GO:0005216 | MF | 0.000106 | 0.000582 | 5 | ion channel activity |
| GO:0004888 | MF | 0.000121 | 0.000612 | 6 | transmembrane signaling receptor activity |
| GO:1901363 | MF | 0.000463 | 0.002184 | 4 | heterocyclic compound binding |
| GO:0008237 | MF | 0.002265 | 0.009965 | 4 | metallopeptidase activity |
| GO:0004175 | MF | 0.012418 | 0.049976 | 3 | endopeptidase activity |
| GO:0004540 | MF | 0.014387 | 0.049976 | 2 | ribonuclease activity |
| GO:0033932 | MF | 0.014387 | 0.049976 | 2 | 1,3-alpha-L-fucosidase activity |
| GO:0046920 | MF | 0.014387 | 0.049976 | 2 | alpha-(1->3)-fucosyltransferase activity |
| GO:0006278 | BP | 4.50E-60 | 4.46E-58 | 47 | RNA-dependent DNA biosynthetic process |
| GO:0090305 | BP | 2.94E-35 | 1.45E-33 | 34 | nucleic acid phosphodiester bond hydrolysis |
| GO:0015074 | BP | 5.54E-23 | 1.83E-21 | 30 | DNA integration |
| GO:0006508 | BP | 2.57E-14 | 6.36E-13 | 31 | proteolysis |
| GO:0090502 | BP | 1.56E-10 | 3.08E-09 | 11 | RNA phosphodiester bond hydrolysis, endonucleolytic |
| GO:0044826 | BP | 6.96E-10 | 1.15E-08 | 7 | viral genome integration into host DNA |
| GO:0016043 | BP | 1.48E-09 | 1.83E-08 | 7 | cellular component organization |
| GO:0075713 | BP | 1.48E-09 | 1.83E-08 | 7 | establishment of integrated proviral latency |
| GO:0006310 | BP | 7.68E-09 | 8.45E-08 | 11 | DNA recombination |
| GO:0046718 | BP | 1.57E-08 | 1.56E-07 | 7 | viral entry into host cell |
| GO:0010467 | BP | 2.17E-08 | 1.95E-07 | 6 | gene expression |
| GO:0000244 | BP | 6.90E-06 | 5.69E-05 | 5 | spliceosomal tri-snRNP complex assembly |
| GO:0075732 | BP | 2.71E-05 | 0.000206 | 4 | viral penetration into host nucleus |
| GO:0051252 | BP | 3.59E-05 | 0.000254 | 7 | regulation of RNA metabolic process |
| GO:0016310 | BP | 3.88E-05 | 0.000256 | 12 | phosphorylation |
| GO:0000398 | BP | 0.000314 | 0.00194 | 10 | mRNA splicing, via spliceosome |
| GO:0034654 | BP | 0.001083 | 0.006309 | 3 | nucleobase-containing compound biosynthetic process |
| GO:0006396 | BP | 0.00213 | 0.011713 | 5 | RNA processing |
| GO:0034645 | BP | 0.002289 | 0.011927 | 3 | cellular macromolecule biosynthetic process |
| GO:0044458 | BP | 0.003422 | 0.016939 | 3 | motile cilium assembly |
| GO:0006810 | BP | 0.004096 | 0.019311 | 3 | transport |
| GO:0006811 | BP | 0.005359 | 0.024116 | 5 | ion transport |
| GO:0065007 | BP | 0.006579 | 0.028318 | 3 | biological regulation |
| GO:0044446 | CC | 5.90E-07 | 3.54E-05 | 6 | intracellular organelle part |
| GO:0042025 | CC | 2.92E-05 | 0.000636 | 4 | host cell nucleus |
| GO:0043231 | CC | 3.18E-05 | 0.000636 | 13 | intracellular membrane-bounded organelle |
| GO:0019012 | CC | 4.51E-05 | 0.000677 | 4 | virion |
| GO:0046540 | CC | 0.000201 | 0.002411 | 5 | U4/U6 x U5 tri-snRNP complex |
| GO:0044444 | CC | 0.000842 | 0.008425 | 4 | cytoplasmic part |
| GO:0044425 | CC | 0.001502 | 0.012872 | 3 | membrane part |
| GO:0044424 | CC | 0.006641 | 0.049806 | 4 | intracellular part |
