# Supplementary material for: An improved genome assembly of the fluke Schistosoma japonicum
Source: PLoS Negl Trop Dis. 2019 Aug 7;13(8):e0007612. doi: 10.1371/journal.pntd.0007612 (PMC6685614; doi:10.1371/journal.pntd.0007612)
Supplement: S10 Table — (DOCX) [file pntd.0007612.s015.docx]

S10 Table. Gene ontology (GO) enrichment analysis for expanded gene families of *Schistosoma japonicum*.

| ID | Class | *P* value | Corrected *P* value | Count | Description |
| --- | --- | --- | --- | --- | --- |
| GO:0004190 | MF | 1.87E-19 | 7.49E-18 | 21 | aspartic-type endopeptidase activity |
| GO:0003676 | MF | 1.65E-14 | 3.30E-13 | 32 | nucleic acid binding |
| GO:0004653 | MF | 9.38E-08 | 1.25E-06 | 5 | polypeptide N-acetylgalactosaminyltransferase activity |
| GO:0008270 | MF | 4.04E-06 | 4.04E-05 | 18 | zinc ion binding |
| GO:0008253 | MF | 1.39E-05 | 9.27E-05 | 4 | 5'-nucleotidase activity |
| GO:0019825 | MF | 1.39E-05 | 9.27E-05 | 4 | oxygen binding |
| GO:0004197 | MF | 2.34E-05 | 0.000134 | 6 | cysteine-type endopeptidase activity |
| GO:0004016 | MF | 2.76E-05 | 0.000138 | 4 | adenylate cyclase activity |
| GO:0004383 | MF | 0.000101 | 0.000405 | 4 | guanylate cyclase activity |
| GO:0008378 | MF | 0.000101 | 0.000405 | 4 | galactosyltransferase activity |
| GO:0030246 | MF | 0.000139 | 0.000505 | 5 | carbohydrate binding |
| GO:0016757 | MF | 0.000226 | 0.000752 | 5 | transferase activity, transferring glycosyl groups |
| GO:0033897 | MF | 0.000609 | 0.001873 | 3 | ribonuclease T2 activity |
| GO:0020037 | MF | 0.001926 | 0.005504 | 4 | heme binding |
| GO:0003824 | MF | 0.002388 | 0.006368 | 5 | catalytic activity |
| GO:0046983 | MF | 0.00703 | 0.017576 | 5 | protein dimerization activity |
| GO:0070063 | MF | 0.010792 | 0.025392 | 2 | RNA polymerase binding |
| GO:0000166 | MF | 0.014247 | 0.030784 | 4 | nucleotide binding |
| GO:0017022 | MF | 0.014622 | 0.030784 | 2 | myosin binding |
| GO:0046982 | MF | 0.018136 | 0.036272 | 6 | protein heterodimerization activity |
| GO:0015074 | BP | 4.64E-30 | 2.14E-28 | 28 | DNA integration |
| GO:0006508 | BP | 1.97E-19 | 4.52E-18 | 27 | proteolysis |
| GO:0006486 | BP | 6.48E-08 | 9.93E-07 | 9 | protein glycosylation |
| GO:0050790 | BP | 6.37E-07 | 7.33E-06 | 6 | regulation of catalytic activity |
| GO:0006171 | BP | 7.85E-06 | 7.22E-05 | 4 | cAMP biosynthetic process |
| GO:0009117 | BP | 2.33E-05 | 1.79E-04 | 4 | nucleotide metabolic process |
| GO:0006182 | BP | 3.63E-05 | 0.000239 | 4 | cGMP biosynthetic process |
| GO:0046039 | BP | 5.41E-05 | 0.000311 | 4 | GTP metabolic process |
| GO:0001666 | BP | 0.000781 | 0.003993 | 4 | response to hypoxia |
| GO:0016311 | BP | 0.001283 | 0.005904 | 4 | dephosphorylation |
| GO:0043170 | BP | 0.004049 | 0.01508 | 2 | macromolecule metabolic process |
| GO:0090314 | BP | 0.004049 | 0.01508 | 2 | positive regulation of protein targeting to membrane |
| GO:0006493 | BP | 0.004917 | 0.01508 | 2 | protein O-linked glycosylation |
| GO:0008347 | BP | 0.004917 | 0.01508 | 2 | glial cell migration |
| GO:0034654 | BP | 0.004917 | 0.01508 | 2 | nucleobase-containing compound biosynthetic process |
| GO:0048477 | BP | 0.007869 | 0.021577 | 3 | oogenesis |
| GO:0006144 | BP | 0.007974 | 0.021577 | 4 | purine nucleobase metabolic process |
| GO:0044238 | BP | 0.010395 | 0.026566 | 2 | primary metabolic process |
| GO:0006810 | BP | 0.011707 | 0.028344 | 2 | transport |
| GO:0071456 | BP | 0.01605 | 0.036915 | 2 | cellular response to hypoxia |
| GO:0008286 | BP | 0.022737 | 0.049805 | 2 | insulin receptor signaling pathway |
| GO:0008074 | CC | 9.21E-07 | 2.12E-05 | 4 | guanylate cyclase complex, soluble |
| GO:0000139 | CC | 1.77E-05 | 0.000203 | 9 | Golgi membrane |
| GO:0001650 | CC | 0.001304 | 0.00753 | 3 | fibrillar center |
| GO:0035577 | CC | 0.001668 | 0.00753 | 2 | azurophil granule membrane |
| GO:0035579 | CC | 0.001668 | 0.00753 | 2 | specific granule membrane |
| GO:0005576 | CC | 0.001964 | 0.00753 | 6 | extracellular region |
| GO:0090575 | CC | 0.003816 | 0.012538 | 2 | RNA polymerase II transcription factor complex |
| GO:0005794 | CC | 0.008052 | 0.02268 | 6 | Golgi apparatus |
| GO:0005667 | CC | 0.008875 | 0.02268 | 5 | transcription factor complex |
